# Perioperative factors related to postoperative mechanical ventilation duration after thoracic surgery requiring one-lung ventilation: a retrospective cohort study

**DOI:** 10.62838/jccm-2026-0044

**Published:** 2026-10-30

**Authors:** Raluca Stefania Fodor, Irina Saplacan, Bianca Liana Grigorescu, Ionut Branea, Leonard Azamfirei

**Affiliations:** Department of Anesthesiology and Intensive Care Medicine, George Emil Palade University of Medicine, Pharmacy, Science and Technology of Târgu Mureș,Romania.; Doctoral School of Medicine and Pharmacy, George Emil Palade University of Medicine, Pharmacy, Science and Technology of Targu Mures,Romania

**Keywords:** one-lung ventilation, thoracic surgery, postoperative mechanical ventilation, ARISCAT, P/F ratio, postoperative pulmonary complications

## Abstract

**Background:**

One-lung ventilation (OLV) is essential for thoracic surgery but may contribute to perioperative gas-exchange impairment and delayed respiratory recovery. Postoperative mechanical ventilation may serve as a pragmatic marker of early respiratory recovery and ICU resource use after thoracic procedures.

**Aim:**

To explore the relationship between a simple set of clinically available perioperative variables and postoperative mechanical ventilation duration after elective thoracic surgery requiring OLV.

**Methods:**

This single-center retrospective observational cohort study included adult patients undergoing elective thoracic surgery requiring OLV. The primary outcome was the time from ICU admission to successful extubation, defined as the absence of reintubation within 48 hours. The multivariable linear regression model of log-transformed ventilation duration included ARISCAT score, OLV duration, intraoperative P/F ratio, and surgical approach.

**Results:**

Ninety-seven patients had complete data for the main multivariable analysis, and no patient required reintubation within 48 hours after extubation. The overall model was statistically significant (F (4,92) = 3.85, R^2^ = 0.1435, adjusted R^2^ = 0.1063, p = 0.0061). OLV duration showed the strongest hypothesis-generating trend: each additional 30 minutes of OLV corresponded to an estimated 8.4% longer postoperative ventilation duration (95% CI, −1.1% to +18.9%, p = 0.082). ARISCAT score, P/F ratio, and thoracotomy versus VATS showed no statistically significant relationship with ventilation duration.

**Conclusions:**

In this retrospective cohort, commonly available perioperative variables explained only a small proportion of the variability in postoperative ventilation duration, and no individual variable demonstrated an independent statistically significant relationship with the outcome. The non-significant trend observed for OLV duration should be considered hypothesis-generating and evaluated in larger cohorts.

## Introduction

One-lung ventilation (OLV) is an essential component of thoracic anesthesia, allowing adequate surgical exposure during pulmonary and pleural procedures. However, OLV imposes non-physiological stress by reducing the ventilated lung volume and increasing ventilation/perfusion mismatch, intrapulmonary shunt, and the risk of impaired oxygenation. These changes may have a greater impact on patients with limited pulmonary reserve or an increased perioperative inflammatory burden [1, 2].

Postoperative pulmonary complications (PPCs) are frequent after major surgery and remain important determinants of postoperative morbidity, ICU admission, prolonged hospital stay, and healthcare resource use. In thoracic surgery, the specific ventilatory requirements of OLV may contribute to postoperative respiratory impairment through atelectasis, barotrauma, volutrauma, and inflammatory lung injury, particularly when high inspired oxygen fractions, low or absent positive endexpiratory pressures, or increased airway pressures are required [3, 4].

Postoperative respiratory recovery after thoracic surgery is clinically important, but respiratory outcomes are often reported as composite PPC endpoints. These composite endpoints may not fully capture the actual burden of postoperative respiratory support. In this setting, postoperative mechanical ventilation duration may provide a pragmatic measure of the interval to respiratory recovery and ICU resource use; however, when all patients are routinely transferred intubated, this interval also reflects emergence from anesthesia, neuromuscular recovery, local extubation workflow, staffing, and other organizational factors [2, 4, 5].

Risk scores such as ARISCAT estimate preoperative pulmonary risk but do not directly quantify postoperative ventilation time. In patients undergoing thoracic surgery with OLV, delayed extubation may result from interactions among baseline respiratory vulnerability, surgical invasiveness, duration of OLV, intraoperative oxygenation impairment, and systemic inflammatory burden [6,7].

Therefore, this study aimed to explore the relationship between a simple set of clinically available perioperative variables - preoperative pulmonary risk assessed by the ARISCAT score, duration of OLV, intraoperative oxygenation impairment assessed by the P/F ratio, and surgical approach – and postoperative mechanical ventilation duration after elective thoracic surgery requiring OLV. Preoperative CRP was examined separately due to limited data availability.

## Materials and Methods

### Study design and population

This was a single-center retrospective observational cohort study of adult patients scheduled for elective thoracic surgery requiring OLV. Patients were consecutively included between January 2024 and March 2026 during hospitalization in the Anesthesia and Intensive Care Department of the Târgu Mureș Emergency Clinical County Hospital, Mureș County, Romania. Of 103 patients assessed for eligibility, six were excluded from the complete-case primary analysis because no valid arterial blood gas sample obtained during OLV was available, preventing calculation of the P/F ratio required for the prespecified model. The final analytical cohort therefore comprised 97 patients.

The study was approved by the hospital Ethics Committee (approval no. 35163/12.01.2024). GDPR requirements were observed, and all collected data were used for research purposes only.

Eligible patients were adults aged 18 years or older undergoing elective thoracic surgery requiring OLV. Patients were excluded if surgery was performed as an emergency procedure, if relevant preoperative respiratory or hemodynamic instability was present, or if the institutional OLV protocol could not be applied. Pre-operative respiratory instability was considered present when invasive or non-invasive ventilatory support was required before surgery or when SpO_2_ remained <90% despite supplemental oxygen. Hemodynamic instability was defined as the need for vasopressor or inotropic support, or a mean arterial pressure <65 mmHg despite initial resuscitation. Patients with active infection requiring antimicrobial treatment, fever ≥38°C with supporting clinical or laboratory findings, acute inflammatory disease, recent systemic corticosteroid or immunosuppressive treatment, missing variables for the prespecified model, or no valid arterial blood gas sample 30 ± 10 minutes after OLV initiation were also excluded.

### Anesthetic and ventilatory management

All patients received general anesthesia with lung isolation using a double-lumen tube. Anesthesia was maintained with sevoflurane according to institutional practice. Surgical procedures were classified as thoracotomy or thoracoscopy/video-assisted thoracoscopic surgery (VATS).

During OLV, protective ventilation was applied with a tidal volume of 6 mL/kg of predicted body weight and a positive end-expiratory pressure of 5 cmH2O. FiO2 was adjusted according to oxygenation status, targeting an SpO2 of 92–94% whenever possible.

During the study period, transfer to the ICU while still intubated was the routine institutional pathway after thoracic surgery requiring OLV and did not, by itself, indicate intraoperative respiratory failure. Emergence, reversal of neuromuscular blockade, and extubation-readiness assessment were completed in the ICU. Extubation criteria included: an awake or easily arousable patient able to follow simple commands; SpO_2_ ≥92% with FiO_2_ ≤0.40 or P/F ratio ≥150 mmHg; respiratory rate 8–30 breaths/min with adequate spontaneous tidal volume (approximately ≥5 mL/kg predicted body weight) and no signs of respiratory distress; core temperature ≥36°C; hemodynamic stability with mean arterial pressure ≥65 mmHg and no escalating vasoactive support. Patients who did not meet these criteria remained mechanically ventilated until extubation and were reassessed. Consequently, the measured interval to extubation reflects both patient recovery and the local postoperative care process, including awakening time and ICU workflow.

### Data collection

Demographic, clinical, surgical, laboratory, and arterial blood gas data were collected from institutional medical records. Recorded variables included age, sex, body mass index (BMI), American Society of Anesthesiologists (ASA) physical status, surgical approach, ARISCAT score, SPORC score, OLV duration, intraoperative P/F ratio, postoperative mechanical ventilation duration, and reintubation within 48 hours after extubation.

Preoperative inflammatory status was assessed using serum C-reactive protein (CRP) when available. CRP was not included in the main multivariable model because complete CRP data were available only in a subgroup of patients.

Arterial blood gas samples were obtained 30 minutes after the initiation of OLV and were used for intra-operative gas-exchange assessment. The P/F ratio was calculated as arterial oxygen partial pressre divided by the inspired oxygen fraction at the time of sampling.

### Outcome

The primary outcome was the duration of postoperative mechanical ventilation, expressed in minutes and defined as the time from ICU admission to successful extubation. Successful extubation was defined as the absence of reintubation within 48 hours after extubation. No patient required reintubation during this period; therefore, the time to first and successful extubation were identical in this cohort. No patients required tracheostomy, died before extubation, or were transferred while still intubated. Reintubation events, if present, were not included in the primary endpoint.

### Statistical analysis

Statistical analyses were performed using GraphPad Prism. Continuous variables are presented as median with interquartile range, and categorical variables as number and percentage. Because postoperative ventilation duration was right-skewed, it was natural-log transformed before regression analysis. The main analysis used a complete-case approach without imputation and included ARISCAT score, OLV duration, P/F ratio, and surgical approach. Model variables were scaled for clinical interpretation: ARISCAT score per 10-point increase, one-lung ventilation duration per 30-minute increase, and P/F ratio per 50-mmHg increase. VATS was coded as 0 and thoracotomy as 1. Separate univariable regression models were used for P/F ratio and CRP. For the CRP analysis, both CRP and postoperative ventilation duration were natural log transformed. Results are reported as β coefficients with 95% confidence intervals and p-values. A two-sided p-value <0.05 was considered statistically significant. The global F test was interpreted as a test of the model, not as evidence that any individual variable was independently related to the outcome.

SPORC was reported descriptively but excluded from the prespecified model because it was developed to estimate the risk of postoperative respiratory failure/mechanical ventilation and therefore conceptually overlapped with the study endpoint. Percent changes were calculated from log-linear coefficients as (eβ − 1) × 100.

## Results

### Literature Search

Of 103 patients assessed for eligibility, six lacked a valid intraoperative arterial blood gas sample during OLV and were excluded because the P/F ratio required for the prespecified model could not be calculated. The primary complete-case analysis therefore included 97 patients. Within this analytical cohort, no data were missing for age, sex, BMI, ASA physical status, ARISCAT, SPORC, surgical approach, OLV duration, P/F ratio, time to successful extubation, or 48-hour reintubation status. CRP was available in 34 patients and missing in 63 patients; it was therefore analyzed separately. Patient selection is shown in Figure 1.

**Fig. 1.**
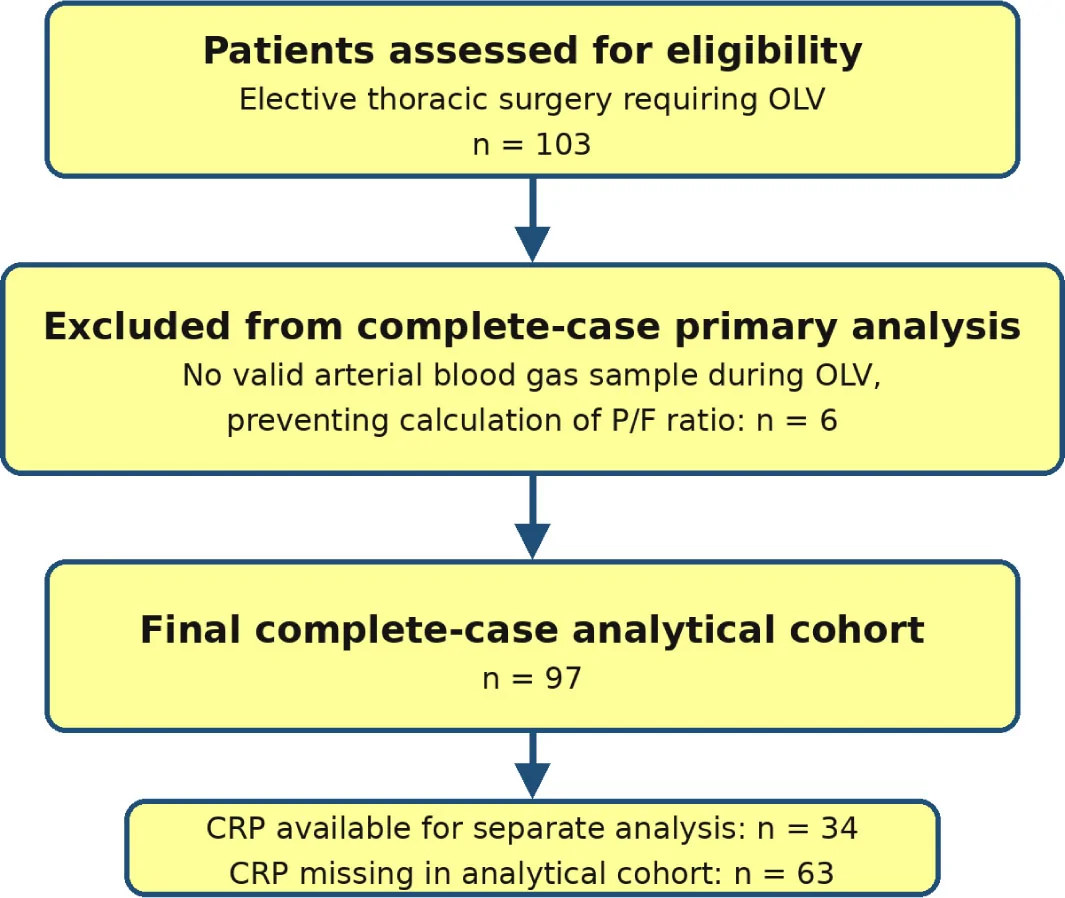
Patient selection and availability of data for the primary complete-case analysis and the separate CRP analysis.

Median age was 62 years (IQR 53–69), and 60 patients were male (61.9%). Median BMI was 25.21 kg/m^2^ (IQR 22.46–29.07). Most patients were classified as ASA physical status III or IV. Median ARISCAT score was 50 (IQR 40–51), and median SPORC score was 7 (IQR 6–8). Thoracotomy was performed in 71 patients (73.2%), and VATS in 26 patients (26.8%). Median one-lung ventilation duration was 130 minutes (IQR 70–180), median P/F ratio was 105 (IQR 77–160), and median postoperative ventilation duration was 125 minutes (IQR 60–210). No patient required reintubation within 48 hours. CRP data were available in 34 patients, with a median value of 22.1 (IQR 6.05–70.9).

**Table 1:** Baseline clinical and perioperative characteristics of the analytical cohort (n = 97).

**Variable**	**Analytical cohort (n = 97)**
Age, years [median (IQR)]	62 (53–69)
Male sex (n)	60 (61.9%)
BMI, kg/m^2^ [median (IQR)]	25.21 (22.46–29.07)
ASA physical status II (n)	6 (6.2%)
ASA physical status III (n)	55 (56.7%)
ASA physical status IV (n)	36 (37.1%)
ARISCAT score [median (IQR)]	50 (40–51)
SPORC score [median (IQR)]	7 (6–8)
Thoracotomy procedures (n)	71 (73.2%)
VATS procedures (n)	26 (26.8%)
One-lung ventilation duration, min [median (IQR)]	130 (70–180)
P/F ratio [median (IQR)]	105 (77–160)
Time to successful extubation, min [median (IQR)]	125 (60–210)
CRP, mg/L [median (IQR); n = 34]	22.1 (6.05–70.9)

The global multivariable model test was statistically significant, F (4,92) = 3.85, p = 0.0061. Nevertheless, model performance remained low: R^2^ was 0.1435 and adjusted R^2^ was 0.1063, indicating that the model explained only approximately 10.6% of the variability in log-transformed postoperative ventilation duration after adjustment. Model performance is summarized in Table 2.

**Table 2:** Performance of the main multivariable regression model for postoperative mechanical ventilation duration.

**Statistic**	**Result**	**Interpretation**
Observations	97	Patients with complete data included in the main model
R2	0.1435	Model explains 14.4% of the variation in log-transformed postoperative ventilation duration
Adjusted R2	0.1063	Adjusted explanatory power is 10.6%
F-test p-value	0.0061	Statistically significant global test despite low explanatory performance
F statistics (df1, df2)	3.85 (4, 92)	Global test of the four-variable model

No individual predictor reached conventional statistical significance in the main multivariable model. OLV duration showed the strongest positive signal: each additional 30 minutes of OLV was associated with an estimated 8.4% longer postoperative ventilation duration, although this association did not reach statistical significance (95% CI −1.1% to +18.9%, p = 0.082). Model coefficients are shown in Table 3.

**Table 3:** Variables included in the main multivariable model.

**Model variable**	**Beta**	**% change in postoperative ventilation duration**	**95% CI**	**p-value**
ARISCAT score, per 10 points	0.123	+13.1%	−4.7% to +34.1%	0.156
OLV duration, per 30 minutes	0.081	+8.4%	−1.1% to +18.9%	0.082
P/F ratio, per 50 mmHg increase	−0.058	−5.7%	−16.5% to +6.6%	0.347
Thoracotomy vs VATS	0.173	+18.8%	−20.2% to +77.0%	0.392

In univariable log-transformed regression, P/F ratio showed no statistically significant relationship with postoperative ventilation duration (p = 0.537). In the univariable analysis, P/F ratio was not significantly related to postoperative ventilation duration (p = 0.537), with wide confidence intervals crossing the null.

CRP was analyzed separately because of limited available data. In univariable log-log regression among 34 patients, CRP showed no statistically significant relationship with postoperative ventilation duration. A doubling of CRP corresponded to an estimated 0.13% shorter postoperative ventilation duration (95% CI −3.6% to +3.4%, p = 0.940), a negligible and imprecise estimate that should be interpreted with caution given the small subgroup.

**Table 4:** Univariable log-log regression of CRP and postoperative ventilation duration.

**Statistic**	**Value**
Observations	34
R2	0.00018
Adjusted R2	−0.031
Beta for ln (CRP)	−0.0019
p-value	0.9395
95% CI for beta	−0.0526 to +0.0488

The overall multivariable model was statistically significant, but no individual predictor was statistically significant. OLV duration showed the strongest positive signal toward longer postoperative ventilation duration. P/F ratio was not significantly associated with postoperative ventilation duration in either univariable or multivariable regression. CRP was not associated with postoperative ventilation duration in the subgroup with available data.

## Discussion

In this retrospective cohort of patients undergoing elective thoracic surgery requiring OLV, a simple multivariable model including ARISCAT score, OLV duration, P/F ratio, and surgical approach was statistically significant overall, suggesting that these variables contributed to variation in postoperative mechanical ventilation duration. However, the model explained only a small proportion of outcome variability (adjusted R^2^ approximately 10.6%), and no individual model variable reached statistical significance. A significant global F test indicates that the set of variables improves fit compared with an intercept-only model; it does not establish that any individual variable is independently related to postoperative ventilation duration.

Postoperative ventilation duration in this cohort should be interpreted as a combined patient- and process-dependent outcome. Because routine institutional practice was to transfer all patients intubated to the ICU, the measured interval to successful extubation included respiratory recovery but also emergence from anesthesia, reversal and assessment of neuromuscular blockade, readiness for extubation, ICU staffing, and local workflow. The median interval of 125 minutes therefore cannot be considered a pure measure of postoperative pulmonary dysfunction. Nevertheless, no patient required reintubation within 48 hours, indicating that all extubation met the prespecified definition of successful extubation. More broadly, early recovery after thoracic surgery is likely multifactorial and reflects baseline pulmonary reserve, surgical trauma, OLV exposure, gas exchange, pain, atelectasis, residual anesthetic effects, neuromuscular recovery, and institutional extubation practice [8, 9]. Therefore, postoperative ventilation duration reflects not only patient recovery, but also local postoperative care and extubation practices. This should be considered when comparing results across centers.

OLV duration showed the strongest signal in the multivariable model. Each additional 30 minutes of OLV was associated with an estimated 8.4% longer postoperative ventilation duration, but the confidence interval crossed the null, and the result was not statistically significant (p = 0.082). It should therefore be described only as a non-significant trend or hypothesis-generating observation, not as a demonstrated predictor. This direction of effect is consistent with previous studies linking longer OLV exposure and intraoperative ventilatory factors with postoperative pulmonary complications in thoracic surgical populations [2,8,9]. Lai et al. found that longer OLV duration was associated with an increased risk of PPCs after McKeown esophagectomy, while Piccioni et al. reported postoperative pulmonary complications after major lung resection in relation to OLV ventilatory strategy [8,9]. Although our endpoint was postoperative ventilation duration rather than PPCs, these findings support the hypothesis that prolonged OLV may contribute to delayed postoperative respiratory recovery.

ARISCAT score corresponded to an estimated 13.1% longer postoperative ventilation duration per 10-point increase, but this association was not statistically significant. This result should be interpreted in the context of the ARISCAT score’s original purpose. ARISCAT was developed to predict postoperative pulmonary complications in broad surgical populations rather than postoperative ventilation duration. Postoperative ventilation duration is a more specific outcome than PPCs and reflects multiple perioperative factors, including respiratory recovery, gas exchange, surgical burden and readiness for extubation according to standardized clinical criteria [10, 11].

COPD and smoking status were available in the dataset and were tested as additional baseline respiratory variables; neither was significantly related to postoperative ventilation duration and adding them did not improve the model.

The P/F ratio showed no statistically significant relationship with postoperative ventilation duration in either univariable or multivariable analysis. In the multivariable model, each 50-mmHg increase corresponded to an estimated 5.7% shorter ventilation duration, but the confidence interval was wide (−16.5% to +6.6%) and crossed the null. Although the P/F ratio is widely used as an oxygenation index, a single intraoperative value during OLV may be affected by timing of measurement, FiO2 administration, recruitment maneuvers, ventilatory settings, surgical manipulation, atelectasis, and anesthetic effects. For this reason, in our cohort the P/F ratio may be more informative when interpreted within a multivariable perioperative model rather than as an isolated predictor of postoperative ventilation duration [12]. Similarly, Douville et al. found that OLV duration, but not time-weighted average FiO2, was independently associated with PPCs after thoracic surgery requiring OLV. Although their endpoint differed from ours, their findings support the interpretation that isolated intraoperative oxygenation-related variables may have limited predictive value when considered outside the broader perioperative context [13].

Surgical approach also showed no statistically significant relationship with postoperative ventilation duration. Thoracotomy corresponded to an estimated 18.8% longer postoperative ventilation duration compared with VATS, but the confidence interval was wide and included the null. The direction of effect is consistent with previous thoracic surgical literature. Menna et al. reported lower wound-site inflammatory changes after VATS compared with thoracotomy, while Ng and Lau described a reduced postoperative inflammatory response and better preservation of immune function after minimally invasive lung resection [14,15]. However, the present sample was not sufficiently powered to demonstrate a relationship with time to successful extubation.

CRP was analyzed separately because complete data were available for fewer patients. The estimated effect was negligible and not statistically significant, indicating that CRP may reflect the inflammatory burden of surgery or comorbidity but does not directly measure respiratory mechanics or extubation readiness.

Postoperative ventilation duration after thoracic surgery appears to be influenced by several interacting perioperative factors. The modest explanatory power of the model indicates that delayed respiratory recovery cannot be fully explained by ARISCAT score, OLV duration, P/F ratio, and surgical approach alone. Future studies should use a standardized extubation pathway and collect variables that were unavailable in the present database, including COPD, smoking status, pulmonary function, type and extent of resection, total operative duration, blood loss, fluid balance, analgesic technique and opioid exposure, quantitative neuromuscular monitoring and reversal, longitudinal FiO_2_ and PEEP, plateau and driving pressures, and hypoxemia burden [16,17].

## Limitations

Several limitations should be considered. First, this was a single-center retrospective study with a modest sample size, limiting statistical power and external validity. Second, all patients were routinely transferred intubated to the ICU; therefore, time to successful extubation was influenced by emergence, quantitative neuromuscular recovery assessment, local extubation practice, staffing, and organizational factors in addition to respiratory recovery. Third, although no reintubations occurred within 48 hours and all extubations met the study definition of successful extubation, other clinically important postoperative respiratory outcomes were not systematically captured; therefore, relationships with PPCs, non-invasive respiratory support, ICU stay, or hospital stay could not be assessed. Fourth, important clinical and intraoperative variables were not included, such as pulmonary function tests, COPD status, smoking history, type and extent of re-section, duration of surgery, blood loss, intraoperative fluid balance, postoperative analgesia, neuromuscular blockade and reversal, and postoperative pain control. Fifth, CRP was available only in a subgroup of patients, limiting the interpretation of the inflammatory analysis. Finally, a single arterial blood gas measurement 30 minutes after OLV initiation may not represent the full intraoperative oxygenation course.

## Conclusions

In this retrospective cohort, a four-variable model comprising ARISCAT score, OLV duration, P/F ratio, and surgical approach had low explanatory performance for postoperative mechanical ventilation duration among patients undergoing elective thoracic surgery requiring OLV. Although the global model test was statistically significant, no individual variable demonstrated a statistically significant independent relationship with the outcome. The finding for OLV duration represents only a non-significant, hypothesis-generating trend. Larger studies with standardized extubation criteria, complete perioperative respiratory data, and clinically meaningful postoperative outcomes are required. Future prospective multicenter studies with standardized extubation protocols and more complete perioperative data collection are needed to confirm these findings.
